# Preclinical evaluation of the ROR1‐targeting antibody–drug conjugates zilovertamab vedotin and VLS‐211 against B‐cell ALL patient‐derived xenografts

**DOI:** 10.1002/hem3.70438

**Published:** 2026-07-22

**Authors:** Christopher M. Smith, Ben Watts, Kathryn Evans, Hansen Kosasih, Chelsea Mayoh, Stephen W. Erickson, Eric J. Earley, Steven Neuhauser, Timothy M. Stearns, Vivek M. Philip, Jeffrey H. Chuang, Charles E. de Bock, Edward P. Bowman, Katti A. Jessen, Emily L. Jocoy, Carol J. Bult, Beverly A. Teicher, Malcolm A. Smith, Richard B. Lock

**Affiliations:** ^1^ Children's Cancer Institute, Lowy Cancer Research Centre, School of Clinical Medicine, UNSW Medicine & Health UNSW Centre for Childhood Cancer Research, UNSW Sydney Sydney New South Wales Australia; ^2^ RTI International, Research Triangle Park North Carolina USA; ^3^ The Jackson Laboratory for Mammalian Genetics Bar Harbor Maine USA; ^4^ The Jackson Laboratory for Genomic Medicine Farmington Connecticut USA; ^5^ Department of Oncology Merck & Co. Inc. Rahway New Jersey USA; ^6^ Formerly VelosBio Inc. San Diego California USA; ^7^ The Jackson Laboratory Sacramento California USA; ^8^ National Cancer Institute Bethesda Maryland USA

## Abstract

Although survival rates for acute lymphoblastic leukemia (ALL) have improved dramatically over the past 60 years, outcomes vary across different molecular subtypes and risk categories, and a significant number of patients remain difficult to treat. ROR1 is a receptor tyrosine kinase expressed in B‐cell ALL, particularly in patients with *TCF3*::*PBX1* gene rearrangements. ROR1 has served as the target of several immune‐based therapies including antibody–drug conjugates (ADCs), bispecific T‐cell engagers, and CAR T‐cell therapies. Here, we evaluated the ROR1‐targeting ADCs zilovertamab vedotin (ZV, previously known as MK‐2140 or VLS‐101) and VLS‐211 in vivo against a panel of ALL patient‐derived xenografts with variable ROR1 expression. Both agents showed modest activity, which was dependent on ROR1 expression. Notably, we identified pediatric patients with *TCF3*::*HLF* gene fusions, which is a highly chemoresistant ALL subtype, as having some of the highest ROR1 expression in pediatric ALL and show that this subtype is susceptible to targeting ROR1 via ADC‐based therapy. Given that ZV has a favorable toxicity profile in patients with hematological malignancies, it may have some utility in the treatment of very high‐risk pediatric B‐ALL, particularly cases with *TCF3*::*HLF* gene fusions.

## INTRODUCTION

Acute lymphoblastic leukemia (ALL) accounts for approximately 25% of all pediatric cancer diagnoses.^1^ Over the past 60 years, patient outcomes have improved dramatically, with 5‐year survival rates reaching 92% for children (ages 0–14 years) and 77% for adolescents (ages 15–19 years).^2^ However, ALL is a heterogenous disease characterized by many distinct molecular subtypes, some of which remain very challenging to treat.^3^, ^4^

Receptor tyrosine kinase‐like orphan receptor 1 (ROR1, NTRKR1) is a receptor tyrosine kinase expressed in several blood cancers, including chronic lymphocytic leukemia and ALL.^5^ Upon binding with its ligand Wnt5a, ROR1 can mediate cell growth and survival through signaling of the PI3K/AKT, NF‐κB, and MEK/ERK pathways.^6^, ^7^, ^8^ ROR1 is expressed on the cell surface of embryonic stem cells, but is lost upon differentiation and is virtually absent in adult tissues.^9^, ^10^ In B‐cell ALL (B‐ALL), ROR1 expression has been reported in up to 45% of pediatric ALL patients, most notably in patients harboring *TCF3*::*PBX1* rearrangements, where it cooperates with pre‐BCR signaling to promote cell survival.^11^, ^12^, ^13^ The key role and tumor‐specific expression of ROR1 in various malignancies have led to the ongoing development of ROR1‐targeting therapies, including monoclonal antibodies, bispecific T‐cell engagers (BiTEs), antibody–drug conjugates (ADCs), and CAR T‐cell therapy.^7^, ^14^, ^15^, ^16^

Zilovertamab vedotin (ZV, previously known as MK‐2140 or VLS‐101) is an ADC comprised of the ROR1‐targeting monoclonal antibody UC‐961 (cirmtuzumab) and an antimicrotubule cytotoxin monomethyl auristatin E (MMAE) payload.^17^ VLS‐211 is an investigational ADC comprised of UC‐961 and the anthracycline PNU‐159682 (PNU) payload.^18^ Both ZV and VLS‐211 bind to membrane‐bound ROR1, leading to rapid internalization and lysosomal‐mediated cleavage, which releases the cytotoxic payload. ZV has shown efficacy in vivo against Richter syndrome^17^ and mantle cell lymphoma^19^ patient‐derived xenografts (PDXs). In clinical testing, ZV has shown a tolerable safety profile, with clinically meaningful activity for patients with diffuse large B‐cell lymphoma (DLBCL) and mantle cell lymphoma.^20^, ^21^, ^22^ ZV administered in combination with chemotherapy is being evaluated in phase 3 clinical trials for patients with DLBCL (NCT06717347 and NCT06890884). A phase 1/2 clinical trial evaluating ZV in pediatric patients with relapsed or refractory B‐ALL, DLBCL/Burkitt lymphoma, neuroblastoma, and Ewing sarcoma was recently initiated (LIGHTBEAM‐U01‐Substudy 01A, ClinicalTrials.gov Identifier NCT06395103).

In this study, we characterized ROR1 expression in pediatric B‐ALL patients and PDXs, and evaluated the single‐agent preclinical activity of ZV and VLS‐211 against a panel of pediatric B‐ALL PDXs in vivo. ROR1 expression was assessed via RNA‐seq and flow cytometry. To characterize the pharmacokinetic (PK) and pharmacodynamic (PD) properties of ZV, plasma levels, ROR1 receptor occupancy, and leukemic burden in multiple organs were assessed over a 168‐hour period following a single administration in NSG mice. Additionally, the activity of ZV was assessed in combination with an induction‐type regimen comprised of vincristine, dexamethasone, and *L*‐asparaginase (VXL) in a PDX with high ROR1 expression.

## MATERIALS AND METHODS

### Establishment of ALL PDXs

All animal experiments received prior approval from the Animal Care and Ethics Committee of UNSW Sydney (Sydney, NSW, Australia). The PDXs used throughout these experiments were previously established in 20–25 g female non‐obese diabetic/severe combined immuno‐deficient (NOD.CB17‐*Prkdc* ^*scid*^/SzJAusb, NOD/SCID) or NOD/SCID/interleukin‐2 receptor γ–negative (NOD.Cg‐*Prkdc* ^*scid*^ *Il2rg* ^*tm1*^Wjl/SzJAusb, NSG) mice (Australian BioResources), as described previously.^23^, ^24^, ^25^ These PDXs are aggressive orthotopic models of the disease, showing high‐level infiltration of the bone marrow (BM) and the spleen, and disseminating to other organs and peripheral blood (PB). The demographic and molecular characteristics of the seven pediatric ALL PDXs used in the in vivo studies are included in Supporting Information S2: Table S1.

### Quantification of *ROR1* messenger RNA (mRNA) expression in ALL PDXs, cell lines, and patients

*ROR1* mRNA expression was assessed in 90 pediatric ALL PDXs via whole‐transcriptome RNA sequencing (RNA‐seq) as previously described.^25^ Raw reads were aligned to the human genome (hg38) using the STAR aligner (v2.7.8a) in basic two‐pass mode.^26^ The Picard toolkit (v2.27.5, http://broadinstitute.github.io/picard) was used to assign read groups, sort reads by genome coordinates, and remove duplicates in the bam files using the tools AddOrReplaceReadGroups, SortSam, and MarkDuplicates, respectively. Gene expression was quantified using RSEM (v1.3.3) with GENCODE v41 gene annotations and reported as transcripts per million reads (TPM).^27^ ROR1 cell surface and mRNA expression were used to guide the selection of PDXs for in vivo experiments. Processed RNA‐seq expression data (as TPM) for the 697, HAL‐01, and NALM‐6 cell lines were downloaded from the National Center of Biotechnology Information (NCBI) Gene Expression Omnibus (GEO) under accession ID GSE168386. Raw RNA‐seq data for cell lines from the Tamai et al data set^28^ were downloaded from NCBI under BioProject accession ID PRJDB18849 and processed using the nf‐core/rnaseq pipeline (v3.21.0) with the human (hg38) genome and GENCODE v41 annotations to generate TPM values. Publicly accessible ALL patient expression data (as TPM) and subtype annotations were downloaded directly from the St Jude Cloud (https://platform.stjude.cloud) or the GDC database using the TCGAbiolinks R package (v2.32.0) for the TARGET study (TARGET‐ALL‐P2). Patient samples from St Jude Cloud were batch‐corrected for library type using the R package sva (v3.58.0) ComBat_seq function, and samples were confirmed to cluster primarily by biological subtype. Selection of TARGET patients included in this study was based on available data for molecular subtypes and fusions from an independent study.^29^ ROR1 TPM in B‐ and T‐ALL patients was compared using the Wilcoxon rank‐sum tests, and effect sizes were reported as rank‐biserial correlations (*r*_rb_).

### Quantification of ROR1 cell surface expression in ALL PDXs

Flow cytometry was used to measure ROR1 cell surface expression. PDX cells were incubated with anti‐human CD45 (HuCD45 BV421, cat #563879; BD Biosciences) and anti‐mouse CD45 (MuCD45 APC, cat #5599864; BD Biosciences) antibodies, along with a PE‐conjugated ROR1 antibody (ROR1‐PE clone 4A5, cat #564474; BD Biosciences). A fluorescence‐minus‐one (FMO) control, lacking the ROR1‐PE antibody, was included for comparison. The mean fluorescence intensity (MFI) was determined from the peak fluorescence channel geometric mean, and the relative fluorescence intensity (RFI) was defined as the ratio of MFIs between cells stained with ROR1‐PE and the FMO control.

#### Analysis of ChIP‐seq and HiChIP data

For ChIP‐seq, raw fastq files were downloaded from the NCBI Sequence Read Archive (SRA) under run IDs SRR10189271 (697 cell line, H3K27ac antibody), SRR10189274 (697 cell line, PBX1 antibody), SRR7408221 (NALM‐6 cell line, H3K27ac antibody), and from the European Nucleotide Archive under run IDs ERR2618839 (HAL‐01 cell line, H3K27ac antibody) and ERR2618827 (HAL‐01 cell line, FLAG antibody). Reads were aligned to the human reference genome (hg19) using bowtie2 (v2.3.5.1), and reads with a mapping quality (MAPQ) score below 30 were removed using samtools (v1.12). Bigwig files for peak visualization were generated from bam files using deeptools (v3.5.6) bamCoverage, normalized by CPM with ignoreDuplicates on. Processed HiChIP data (in bedpe file format) were downloaded from SRA under run IDs SRR27822330 (697 cell line, H3K27ac HiChIP) and SRR27822329 (NALM‐6, H3K27ac HiChIP).

### Tolerability study of ZV, VLS‐211, and sequential treatment of VXL, followed by ZV in NSG mice

ZV, VLS‐211, and UC‐961 were provided by VelosBio Inc. and formulated in phosphate‐buffered saline (PBS). The anti‐mitotic agent MMAE is stably linked to the ROR1‐targeting monoclonal antibody UC‐961 to form the ADC, ZV. The anthracycline payload, PNU, is stably linked to UC‐961 to form the ADC, VLS‐211. Tolerability testing was carried out in naïve female NSG mice aged 10–12 weeks. Mice were treated with either the company‐recommended dose of ZV (5 mg/kg) or VLS‐211 (1 mg/kg), 50% of the company‐recommended dose (ZV, 2.5 mg/kg; VLS‐211, 0.5 mg/kg), a naked antibody control (UC‐961, 5 mg/kg), or vehicle control (PBS). Mice were scheduled for treatment every 4 days via an intravenous (IV) injection for six total treatments at a maximum administered volume of 5 µL/g body weight. Due to observed toxicity (described below), the treatment schedule for all drugs was altered to once a week from the fourth treatment until the end of the treatment period.

In the second tolerability study for the sequential treatment of VXL and ZV, female NSG mice aged 10–12 weeks were first treated with an induction‐type regimen, VXL (vincristine, V, 0.15 mg/kg; dexamethasone, X, 5 mg/kg; *L*‐asparaginase, L, 1250 U/kg), previously optimized in our lab.^30^ Over a 7‐day rotation, VXL was administered via an intraperitoneal (IP) injection on the first day, followed by four days of XL treatment with a further 2 days of no drug treatment. VXL was administered for the first 2 weeks, and was then followed by ZV administered once weekly for 3 weeks via an IV injection at either 100% of the maximum tolerated dose (5 mg/kg), 75% (3.75 mg/kg), 50% (2.5 mg/kg), or 25% (1.25 mg/kg).

### Efficacy testing of VLS‐211 and ZV alone and in combination with VXL in NSG mice

Efficacy testing was carried out in female NSG mice aged 6–8 weeks. UC‐961, ZV, and VLS‐211 were tested against a panel of seven B‐ALL PDXs (six B‐cell precursor [BCP]‐ALL, one Philadelphia‐positive [Ph^+^]‐ALL). Treatment groups consisted of seven mice (vehicle control, UC‐961) or eight mice (ZV, VLS‐211). In each group, three mice were pre‐assigned for euthanasia upon the occurrence of an event (defined later in this subsection) or at 28 days post‐treatment initiation (E/D28, whichever occurred first) to assess leukemic infiltration of the BM, spleen, and blood via cardiac puncture. The remaining mice were monitored until an event occurred to assess event‐free survival (EFS). Mice assigned to the E/D28 group were included in the EFS calculation if an event occurred before Day 28.

Prior to treatment, mice were inoculated with PDX cells (2–5 × 10^6^ cells per mouse) via a lateral tail vein injection. Engraftment was assessed by weekly enumeration of the proportion of human CD45^+^ (%huCD45^+^) cells versus the total CD45^+^ cells (human and murine) in the PB. Treatment began when the median %huCD45^+^ cells in the PB of each mouse cohort was at least 1%. Mice were pretreated with the platelet‐activating factor (PAF) inhibitor, CV‐6209 (5 mg/kg via IP injection), 1 h before treatment with either UC‐961, ZV, or VLS‐211. The PAF inhibitor was included to reduce potential hypersensitivity reactions that can occur following treatment with antibodies. UC‐961 (5 mg/kg), ZV (2.5 and 5 mg/kg), and VLS‐211 (0.25 and 0.5 mg/kg) were administered weekly for three weeks via an IV injection.

Additional testing of ZV following VXL treatment was conducted in a single BCP‐ALL PDX model (ALL‐7). VXL (V, 0.15 mg/kg; X, 5 mg/kg; L, 1250 U/kg) was administered via an IP injection for 2 weeks, consistent with the tolerability study dosing regimen. This was followed by weekly IV administration of either vehicle control (PBS) or ZV (5 mg/kg) for 3 consecutive weeks. An additional group was administered a VXL vehicle control (PBS) via IP for the first 2 weeks of treatment, followed by a ZV vehicle control weekly for 3 weeks. Mice were pretreated with CV‐6209 (5 mg/kg) 1 h before receiving ZV or vehicle control.

Events were predefined as 25% huCD45^+^ in the PB, or mice showing signs of leukemia‐related morbidity associated with at least 50% huCD45^+^ in two or more major organs, as assessed by flow cytometry. Time to event was calculated by interpolating between the measurements directly before and after the event, assuming log‐linear growth, and represented with Kaplan–Meier analysis.^31^ Response to treatment was assessed by EFS, differences between the median EFS in drug treated (T) and vehicle control (C) groups (T‐C and T/C), and objective response measures (ORMs) based on stringent clinical criteria. Models were considered responsive (partial response, PR; complete response, CR; and maintained complete response, MCR) or nonresponsive (progressive disease 1/2, PD1/2; stable disease, SD) to treatment based on ORM. Survival differences between treatment arms were analyzed using the Peto–Peto modification of the Gehan–Wilcoxon test, with *p*‐values adjusted for multiple testing using the Benjamini–Hochberg procedure. Organ infiltration (%huCD45^+^) at E/D28 was analyzed by two‐way ANOVA, followed by Tukey's HSD post‐hoc test, with PB, BM, spleen, and cardiac blood analyzed as separate families. For a more detailed explanation of the methodology and definitions, see the Supporting Information S1: Methods.

### PK/PD testing and ROR1 receptor occupancy of ZV in NSG mice

PK/PD profiling, ROR1 receptor occupancy, and leukemic burden were assessed in mice engrafted with the BCP‐ALL PDX ALL‐7, selected due to its high ROR1 expression and sensitivity to ZV treatment. Treatment began when mice were estimated to be 20% huCD45^+^ in the PB. Mice (three per group) received a single dose of ZV (2.5 or 5 mg/kg) and were euthanized at designated time points between 2 and 168 h. Two additional control groups were included: a baseline control (euthanized at 0 h) and a vehicle control (euthanized at 168 h). Mice were pre‐treated with CV‐6209 (5 mg/kg via IP injection) one hour before treatment. Post‐euthanasia, the BM, spleen, and blood from cardiac puncture were harvested from mice.

The concentration of “total human IgG” in mouse plasma (total antibody) of the test article was measured by a bioanalytical method using an ECL‐based immunoassay platform. The concentration of “payload conjugated to antibody” in mouse plasma (conjugated payload) was quantified by affinity capture, on‐cartridge proteolytic release, and liquid chromatography‐tandem mass spectrometry (LC‐MS/MS). Detailed methodologies for the total antibody and conjugated payload assays are included in the Supporting Information S1: Methods.

To measure ROR1 receptor occupancy, blood was incubated with anti‐human CD45 (HuCD45 BV421, cat #563879; BD Biosciences), anti‐mouse CD45 (MuCD45 FITC, cat #553080; BD Biosciences), Zombie NIR Fixable Viability Dye (cat #423106; BioLegend), UC‐961‐PE, and a custom ROR1‐targeting antibody (4A5) conjugated to Alexa Fluor 647 (4A5‐AF647, part #94267; BioLegend), which binds to a different epitope to UC‐961. To establish the threshold for positive staining, an FMO control was used for each antibody. The proportion of cells positive for unsaturated ROR1 (%ROR1_FREE_) was determined using the UC‐961‐PE signal, and the percentage positive for total ROR1 (%ROR1_TOTAL_) was determined using the 4A5‐AF647 signal. Receptor occupancy was then calculated using the equation (1 – %ROR1_FREE_/%ROR1_TOTAL_) * 100.^32^ Leukemic burden was also measured for each group by flow cytometric assessment of %huCD45^+^ cells in blood, spleen, and BM samples.

## RESULTS

### ROR1 mRNA and cell surface expression in pediatric ALL PDXs

**Figure 1:**
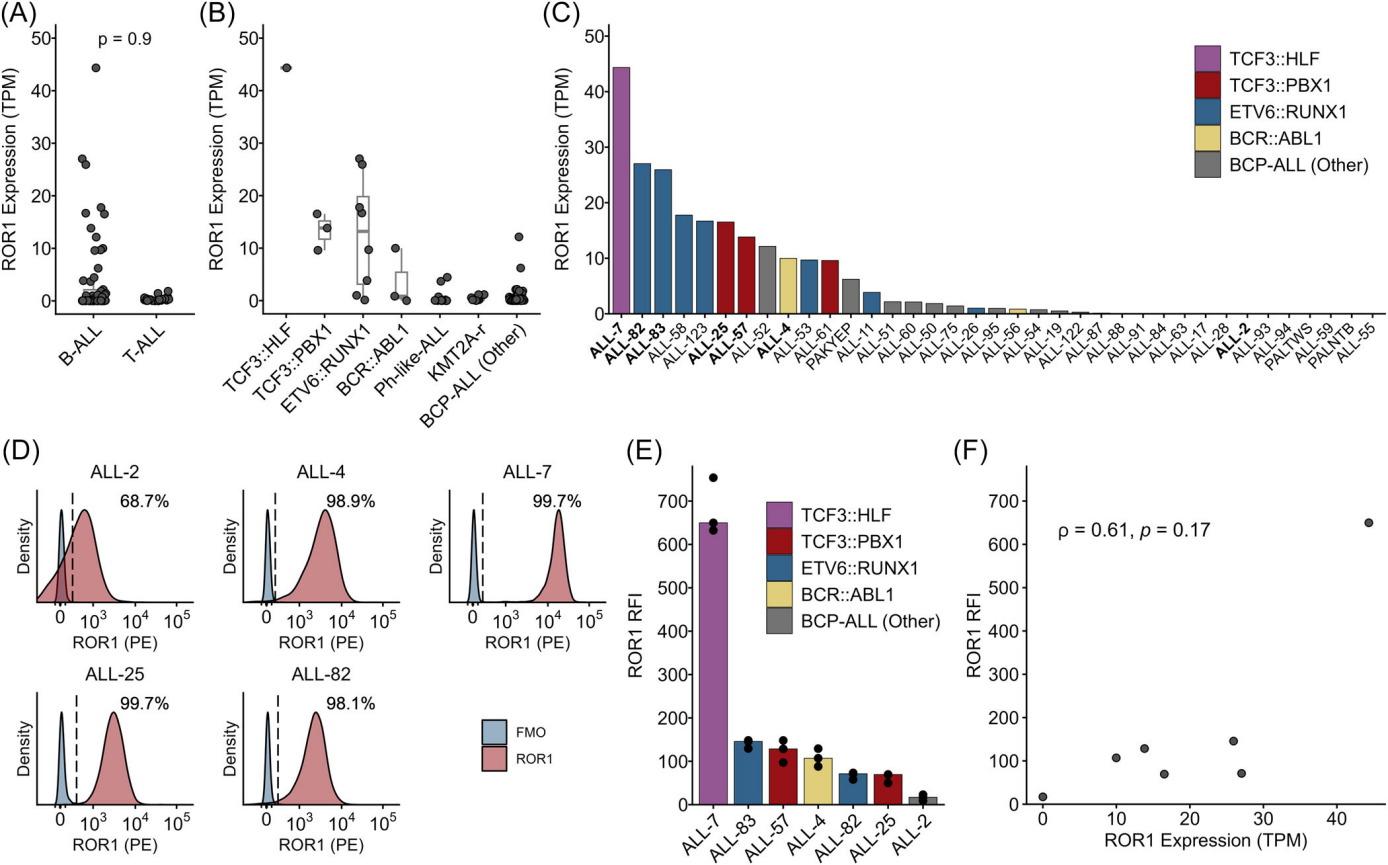
**Expression of ROR1 in pediatric ALL PDXs**. **(A)** *ROR1* mRNA expression in B‐ (*n* = 65) and T‐lineage (*n* = 25) ALL PDXs, compared using the Wilcoxon rank sum test. **(B)** *ROR1* mRNA expression in B‐lineage ALL PDXs (*n* = 65) with known driver fusions or molecular subtypes. **(C)** *ROR1* mRNA expression in individual B‐lineage ALL PDXs (*n* = 1). PDXs used in the efficacy studies are shown in bold. **(A–C)** mRNA expression is measured using transcripts per million (TPM). **(D)** Representative density plots showing ROR1 cell surface expression in leukemia cells from 5 PDXs, determined by flow cytometry. Cells stained with ROR1‐PE (red) or a fluorescence minus one (FMO) control (blue) are shown. The dotted line demarcates the minimum threshold for ROR1^+^ positivity, and the percentages indicate the proportion of ROR1^+^ cells. **(E)** ROR1 cell surface expression (RFI) in select PDXs. Black dots show biological replicates (*n* = 3) and bars show the median RFI. **(F)** Correlation of *ROR1* mRNA and RFI. ρ, Spearman's correlation coefficient; P, P‐value.

The molecular characterization of 90 pediatric ALL PDXs has been previously reported by our group, representing broad subtypes of ALL from both B‐ and T‐cell lineages.^25^ *ROR1* mRNA expression ranged from 0 to 44.3 TPM, with no significant difference between B‐ and T‐cell lineages (P = 0.9) and a negligible effect size (rank‐biserial correlation *r*_rb_ = 0.017, 95% CI [−0.25, 0.28], Figure 1A). However, all PDXs with high expression of *ROR1* belonged to the B‐lineage, with many harboring characteristic driver fusion genes, including *TCF3*::*HLF* (ALL‐7), which had the highest mRNA expression (44.3 TPM), *TCF3*::*PBX1* (ALL‐25, ALL‐57, ALL‐61), *ETV6*::*RUNX1* (ALL‐82, ALL‐83, ALL‐58, ALL‐123, ALL‐53, ALL‐11), and *BCR*::*ABL1* (ALL‐4) fusions (Figure 1B,C). ALL‐82 (27.0 TPM) and ALL‐83 (25.9 TPM) were generated from the same patient at diagnosis and relapse, respectively, and had similar levels of *ROR1* expression.

To investigate *ROR1* expression in a larger cohort of primary ALL samples, we interrogated public databases from the St. Jude Cloud (Supporting Information S1: Figure S1A) and the TARGET study (Supporting Information S1: Figure S1B). This analysis showed that the median *ROR1* expression was the highest in patients harboring *TCF3*::*HLF* and *TCF3*::*PBX1* fusions, with all patients belonging to these two subtypes expressing *ROR1*. We also identified the MEF2D‐rearranged subtype as having characteristically high expression of *ROR1*, although this subtype is not currently represented in our PDX panel. High *ROR1* expression was evident in most subtypes across the larger St. Jude data set and observations were generally consistent with those from our own PDXs. A panel of seven PDXs was chosen for quantifying cell surface expression via flow cytometry (median RFI 17.0‐649.8, Figure 1D,E, Supporting Information S2: Table S1). Analysis of RNA‐seq and flow cytometry confirmed that ALL‐7 had the highest *ROR1* mRNA (44.3 TPM) and ROR1 cell surface expression (649.8 RFI), with nearly all cells ROR1^+^ (median 99.8%). Furthermore, across the seven PDXs ROR1 RFI showed a positive correlation with *ROR1* mRNA expression (Spearman's *ρ* = 0.61, 95% CI [−0.27, 0.93], P = 0.167, Figure 1F). An exception was the patient‐matched ALL‐82 (diagnosis, 71.1 RFI) and ALL‐83 (relapse, 145.7 RFI), which showed differences in ROR1 cell surface expression that were not evident when comparing mRNA expression.

### Integrated analysis of ROR1 regulatory elements in ALL cell lines

To identify potential regulatory elements driving enhanced *ROR1* expression in *TCF3*::*HLF* and *TCF3*::*PBX1* ALL, we performed an integrated analysis of publicly available RNA‐seq, ChIP‐seq, and HiChIP data in the 697 (*TCF3*::*PBX1*) and HAL‐01 (*TCF3*::*HLF*) cell lines (Supporting Information S1: Figure S2). Analysis of RNA‐seq showed a relatively high expression of *ROR1* in both 697 (5.6 median TPM) and HAL‐01 (5.8 TPM) cell lines compared to NALM‐6 (0.4 TPM), which does not harbor a known driver fusion (Supporting Information S1: Figure S2A). High expression of ROR1 in 697 and HAL‐01 cell lines was confirmed in a second data set (Supporting Information S1: Figure S2B). In the 697 cell line, PBX1 binding was co‐localized with the active enhancer histone mark H3K27ac in a region upstream of the *ROR1* gene (referred to as E1). HiChIP looping was evident between the putative E1 enhancer site and the *ROR1* promoter, supporting a direct role for the TCF3*::*PBX1 fusion protein in regulating *ROR1* expression (Supporting Information S1: Figure S2C). Similarly, in the HAL‐01 cell line expressing a FLAG‐tagged *TCF3*::*HLF* fusion (Supporting Information S1: Figure S2D), a FLAG peak upstream of E1 (labeled E2), overlapped with an H3K27ac peak unique to this cell line, supporting a role for this fusion in directly regulating *ROR1* expression in HAL‐01 cells.

### In vivo efficacy of ZV and VLS‐211 against pediatric ALL PDXs

Prior to investigating the in vivo efficacy of ZV and VLS‐211 against pediatric ALL PDXs, a tolerability study to determine the maximum tolerated doses for both ADCs was undertaken in naïve NSG mice. Both ZV (at 2.5 and 5 mg/kg) and the UC‐961 antibody alone (5 mg/kg) were well tolerated on the tested doses, causing no signs of toxicity, as measured by changes in weight/appearance/behavior, or a major shift in hematology or blood biochemistry parameters outside of normal ranges (Supporting Information S1: Figure S3A,B and Supporting Information S2: Tables S2 and 3). In contrast, VLS‐211 was poorly tolerated at both doses tested (0.5 and 1 mg/kg), causing weight loss, with one mouse in the highest dose group (1 mg/kg) being euthanized after three treatments (9 days posttreatment initiation) due to significant weight loss (>20%) from baseline (Supporting Information S1: Figure S3C,D and Supporting Information S2: Tables S2 and 3). The remaining mice in the 1 mg/kg VLS‐211 treatment group were euthanized after meeting humane endpoint criteria after the fourth treatment (14 days posttreatment initiation), despite a recovery in weight. After the fourth treatment, the schedules for ZV and VLS‐211 were changed from once every 4 days to once every week for the remainder of the treatment period. Despite this adjustment, two of four mice in the 0.5 mg/kg VLS‐211 group were euthanized due to >20% weight loss and one mouse was euthanized after meeting humane endpoint criteria following the completion of treatment. As a result, the treatment schedules for all ADCs were altered to once per week for 3 weeks and the doses of VLS‐211 were adjusted to 0.25 mg/kg and 0.5 mg/kg for efficacy testing.

**Figure 2:**
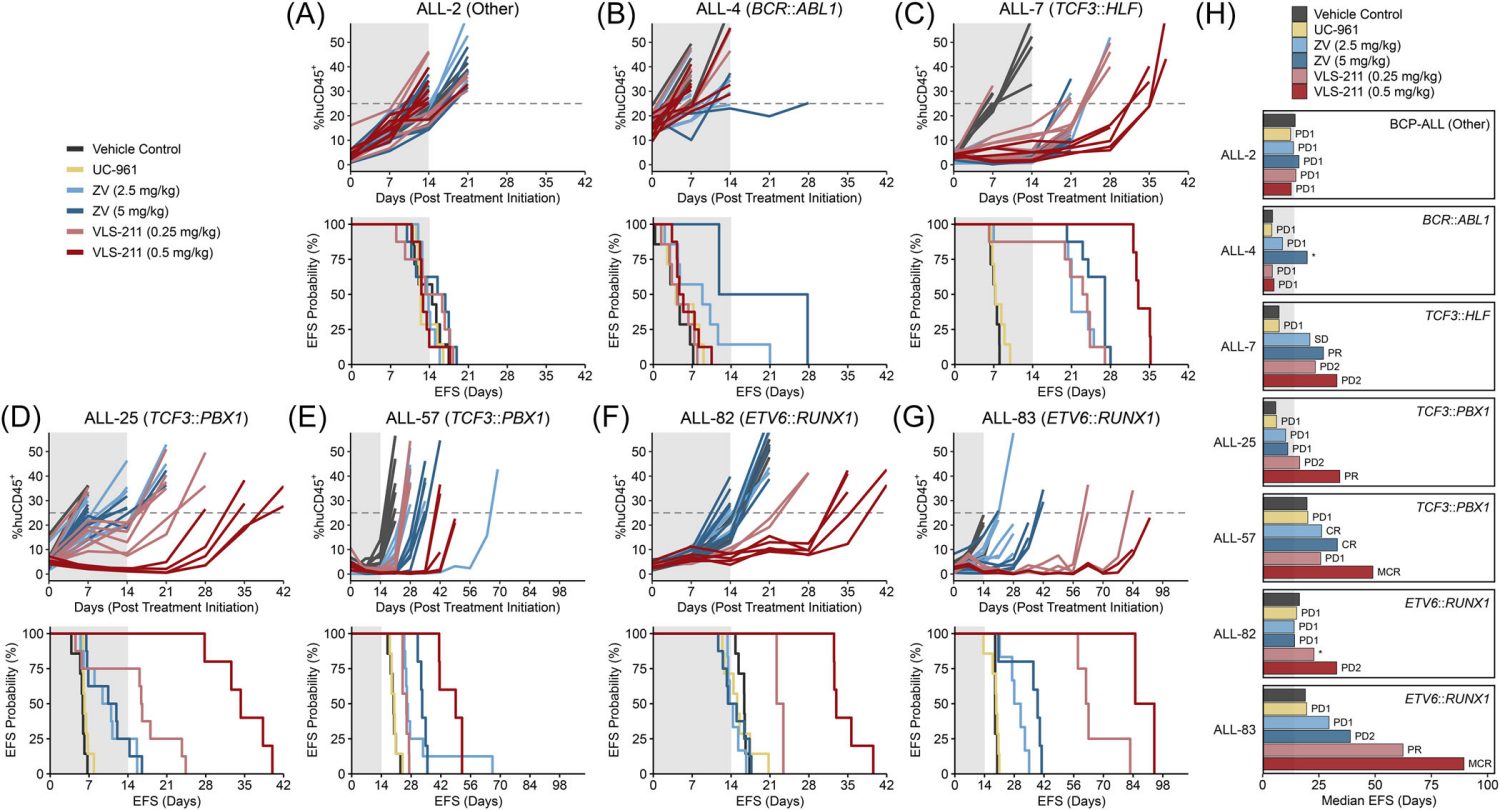
**In vivo efficacy of ZV and VLS‐211 against a panel of pediatric ALL PDXs**. Leukemia burden was measured by enumeration of human CD45^+^ cells (%huCD45^+^) in the PB over time, with treatment commencing when the %huCD45^+^ was greater than a median of 1% for each PDX. The shaded area represents the treatment window and the dotted horizontal line shows the threshold for an event (25% huCD45^+^ cells). **(A–G)** Engraftment (top) and Kaplan–Meier survival (bottom) plots for ALL‐2 **(A)**, ALL‐4 **(B)**, ALL‐7 **(C)**, ALL‐25 **(D)**, ALL‐57 **(E)**, ALL‐82 **(F)**, and ALL‐83 **(G)** after treatment with vehicle control (black), UC‐961 (yellow), ZV (light/dark blue), or VLS‐211 (pink/red). **(H)** Swimmer plots summarizing mouse median EFS in days after treatment initiation and median ORM, shown at the end of each bar. CR, complete response; MCR maintained complete response; PD1, progressive disease 1; PD2, progressive disease 2; PR, partial response; SD, stable disease; *, median ORM not determined, as only two mice were evaluable.

A panel of 7 PDXs including ALL‐2 (Figure 2A), ALL‐4 (Figure 2B), ALL‐7 (Figure 2C), ALL‐25 (Figure 2D), ALL‐57 (Figure 2E), ALL‐82 (Figure 2F), and ALL‐83 (Figure 2G), representing diverse B‐ALL subtypes, were selected for the single‐agent efficacy study. These PDXs were selected for their relatively high ROR1 expression, except for ALL‐2, which was included as a negative control. PDXs were engrafted into NSG mice and treated with either ZV, VLS‐211, UC‐961, or vehicle control. Administration of ZV at 5 mg/kg significantly delayed disease progression in four out of seven PDXs when compared to the vehicle control, including ALL‐7 (T‐C, 19.7 days; T/C, 3.7; adj. P = 0.001), ALL‐25 (T‐C, 5.3 days; T/C, 1.9; adj. P = 0.008), ALL‐57 (T‐C, 13.5 days; T/C, 1.7; adj. P = 0.005), and ALL‐83 (T‐C, 20.0 days; T/C, 2.1; adj. P = 0.010) (Table 1). Significantly delayed disease progression was also observed at the lower dose of 2.5 mg/kg ZV for mice engrafted with ALL‐7 (T‐C, 13.7 days; T/C, 2.9; adj. P = 0.006), ALL‐25 (T‐C, 4.3 days; T/C, 1.7; adj. P = 0.017), ALL‐57 (T‐C, 6.5 days; T/C, 1.3; adj. P = 0.001), and ALL‐83 (T‐C, 10.5 days; T/C, 1.6; adj. P = 0.009). A dose–response relationship was evident within the four PDXs with significantly delayed disease progression after treatment with ZV, each having a greater T‐C and T/C when treated with 5 mg/kg compared to their respective PDXs treated at 2.5 mg/kg. ZV was able to elicit objective responses against two PDXs (Figure 2H and Table 1): ALL‐57 at both dose levels (median ORM, CR) and ALL‐7 at the 5 mg/kg dose (median ORM, PR). All other PDXs treated with ZV scored median ORMs of PD1/PD2 or SD, except for ALL‐4 (treated with 5 mg/kg), which could not be scored because the majority of mice (6/8) were euthanized due to development of mouse lymphomas. Complete summary data for the single‐agent in vivo efficacy experiments are included in Supporting Information S2: Table S4.

**Table 1:** Summary of the in vivo results from the single‐agent efficacy study.

Model	Treatment group	Median EFS	EFS T‐C	EFS T/C	Adjusted P‐value (compared to the vehicle)	Median ORM
ALL‐2	Vehicle	14.5				
	ZV (2.5 mg/kg)	13.7	−0.8	1.0	0.875	PD1
	ZV (5 mg/kg)	16.1	1.7	1.1	0.841	PD1
	VLS‐211 (0.25 mg/kg)	14.8	0.3	1.0	0.841	PD1
	VLS‐211 (0.5 mg/kg)	12.7	−1.8	0.9	0.841	PD1
	UC‐961 (5 mg/kg)	12.4	−2.1	0.9	0.950	PD1
ALL‐4	Vehicle	4.4				
	ZV (2.5 mg/kg)	8.8	4.4	2.0	0.236	PD2
	ZV (5 mg/kg)	19.8	15.4	4.5	0.230	PD2
	VLS‐211 (0.25 mg/kg)	4.3	−0.1	1.0	0.706	PD1
	VLS‐211 (0.5 mg/kg)	5.0	0.6	1.1	0.330	PD1
	UC‐961 (5 mg/kg)	4.1	−0.3	0.9	0.655	PD1
ALL‐7	Vehicle	7.3				
	ZV (2.5 mg/kg)	21.0	13.7	2.9	0.006	SD
	ZV (5 mg/kg)	27.0	19.7	3.7	0.001	PR
	VLS‐211 (0.25 mg/kg)	23.4	16.1	3.2	0.011	PD2
	VLS‐211 (0.5 mg/kg)	33.0	25.7	4.5	0.006	PD2
	UC‐961 (5 mg/kg)	7.3	0.0	1.0	0.364	PD1
ALL‐25	Vehicle	5.9				
	ZV (2.5 mg/kg)	10.2	4.3	1.7	0.017	PD1
	ZV (5 mg/kg)	11.2	5.3	1.9	0.008	PD1
	VLS‐211 (0.25 mg/kg)	16.5	10.6	2.8	0.057	PD2
	VLS‐211 (0.5 mg/kg)	34.3	28.4	5.9	0.008	PR
	UC‐961 (5 mg/kg)	6.2	0.3	1.1	0.057	PD1
ALL‐57	Vehicle	19.7				
	ZV (2.5 mg/kg)	26.2	6.5	1.3	0.001	CR
	ZV (5 mg/kg)	33.2	13.5	1.7	0.005	CR
	VLS‐211 (0.25 mg/kg)	25.8	6.1	1.3	0.002	PD1
	VLS‐211 (0.5 mg/kg)	49.0	29.3	2.5	0.005	MCR
	UC‐961 (5 mg/kg)	20.1	0.3	1.0	0.752	PD1
ALL‐82	Vehicle	16.4				
	ZV (2.5 mg/kg)	13.7	−2.6	0.8	0.047	PD1
	ZV (5 mg/kg)	14.2	−2.2	0.9	0.337	PD1
	VLS‐211 (0.25 mg/kg)	22.8	6.4	1.4	0.092	PD1
	VLS‐211 (0.5 mg/kg)	32.9	16.6	2.0	0.012	PD2
	UC‐961 (5 mg/kg)	15.1	−1.3	0.9	0.417	PD1
ALL‐83	Vehicle	19.0				
	ZV (2.5 mg/kg)	29.5	10.5	1.6	0.009	PD1
	ZV (5 mg/kg)	39.0	20.0	2.1	0.010	PD2
	VLS‐211 (0.25 mg/kg)	62.4	43.4	3.3	0.017	PR
	VLS‐211 (0.5 mg/kg)	89.5	70.5	4.7	0.046	MCR
	UC‐961 (5 mg/kg)	19.6	0.6	1.0	0.468	PD1

*Note*: It includes vehicle control‐, UC‐961‐, ZV‐, and VLS‐211‐treated groups for seven ALL PDXs. For each PDX, EFS of treatment groups was compared using the Peto–Peto modification of the Gehan–Wilcoxon test, and P‐values were adjusted with the Benjamini–Hochberg procedure.

Abbreviations: CR, complete response; EFS, event‐free survival; MCR, maintained complete response; ORM, objective response measure; PD1, progressive disease 1; PD2, progressive disease 2; SD, stable disease; PR, partial response; T‐C, treatment minus control; T/C, treatment divided by control.

VLS‐211 (0.5 mg/kg) elicited significant delays in leukemia progression in five out of seven PDXs, including ALL‐7 (T‐C, 25.7 days; T/C, 4.5; adj. P = 0.006), ALL‐25 (T‐C, 28.4 days; T/C, 5.9; adj. P = 0.008), ALL‐57 (T‐C, 29.3 days; T/C, 2.5; adj. P = 0.005), ALL‐82 (T‐C, 16.6 days; T/C, 2.0; adj. P = 0.012), and ALL‐83 (T‐C, 70.5 days; T/C, 4.7; adj. P = 0.046), compared to the vehicle control (Figure 2 and Table 1). Three PDXs achieved objective responses, with ALL‐57 and ALL‐83 scoring MCRs and ALL‐25 scoring a PR. All remaining PDXs failed to achieve an objective response and were scored as either PD1 or PD2. Administration of VLS‐211 at a lower dose of 0.25 mg/kg caused significant delays in mouse EFS in three out of six evaluable PDXs, including ALL‐7 (T‐C, 16.1 days; T/C, 3.2; adj. P = 0.011), ALL‐57 (T‐C, 6.1 days; T/C, 1.3; adj. P = 0.002), and ALL‐83 (T‐C, 43.4 days; T/C, 3.3; adj. P = 0.017), when compared to the vehicle control. The EFS T‐C ranged from −0.1 to 43.4 days (T/C, 1.0–3.3). ALL‐83 was the only PDX that achieved an objective response (PR), with all other PDXs scoring either PD1 or PD2. The group of mice inoculated with ALL‐82 and treated with 0.25 mg/kg VLS‐211 could not be evaluated for ORM, as six out of eight mice developed hypersensitivity reactions. In general, VLS‐211 at its highest dose (0.5 mg/kg) had the greatest efficacy of the evaluated treatment arms, where delays in disease progression were greater than those observed in ZV for the four PDXs responsive to both agents (ALL‐7, ALL‐25, ALL‐57, and ALL‐83). Furthermore, VLS‐211 at 0.5 mg/kg led to a significant delay in disease progression in ALL‐82, whereas ZV at either dose did not.

Consistent with the expectation that the ROR1‐targeting ADCs would not be effective against PDXs lacking ROR1 expression, we did not observe responses in mice engrafted with ALL‐2 at either of the doses administered for each agent, as measured by significant delays in mouse EFS compared to the vehicle control (Figure 2A), or objective responses (Figure 2H). Notably, treatment with the naked antibody UC‐961 resulted in no significant differences in mouse EFS compared to the vehicle control for any of the PDXs.

Leukemia infiltration in the BM, spleen, and blood via cardiac puncture was assessed by measuring %huCD45^+^ cells (Supporting Information S1: Figures S4 and 5 and Supporting Information S2: Table S5). Mice treated with VLS‐211 (0.5 mg/kg) showed a significant reduction in %huCD45^+^ cells in the BM when compared to vehicle‐treated mice for 5/7 PDXs (ALL‐7, adj. P < 0.001; ALL‐25, adj. P < 0.001; ALL‐57, adj. P = 0.003; ALL‐82, adj. P < 0.001; ALL‐83, adj. P < 0.001). Furthermore, there was a significant reduction of %huCD45^+^ cells for 4/7 PDXs in the blood via cardiac puncture (ALL‐4, adj. P < 0.001; ALL‐57, adj. P < 0.001; ALL‐82, adj. P < 0.001; ALL‐83, adj. P = 0.001) and for 3/7 PDXs in the spleen (ALL‐25, adj. P = 0.029; ALL‐57, adj. P = 0.013; ALL‐83, adj. P < 0.001). Treatment with UC‐961 led to a significant reduction in %huCD45^+^ cells in a single PDX (ALL‐82, adj. P < 0.001). There were no apparent trends indicating a significant decrease in %huCD45^+^ cells for the other treatment groups across the PDX models.

### PK profiling and receptor occupancy of ZV in NSG mice

**Figure 3:**
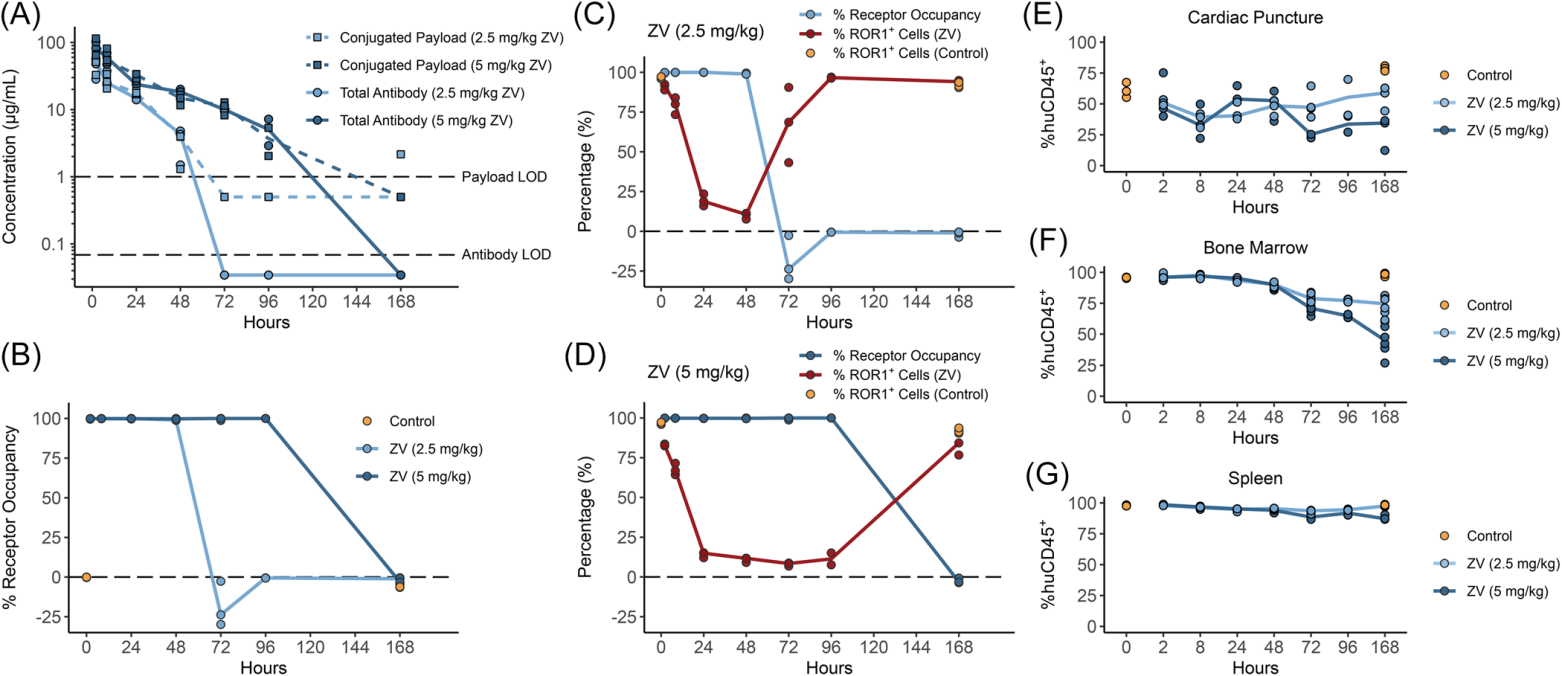
**Pharmacokinetic profile and receptor occupancy in NSG mice after treatment with ZV**. Mice engrafted with the ALL‐7 PDX received a single treatment of ZV at 2.5 mg/kg (light blue) or 5 mg/kg (dark blue), and were euthanized at multiple time points up to 168 h posttreatment (*n* = 3 per treatment per time point). Control groups for the baseline (0 h) and vehicle (168 h) controls are shown in orange. **(A)** Plasma concentration of ZV (conjugated payload, square) and UC‐961 (total antibody, circle) after ZV treatment. LOD, limit of detection. **(B)** Receptor occupancy of ROR1 following ZV treatment. **(C, D)** Proportion of ROR1^+^ cells (red) over 168 h after treatment with 2.5 mg/kg **(C)** or 5 mg/kg **(D)** ZV. The change in receptor occupancy (blue) is reproduced from Figure3B for comparison. **(E–G)** Leukemia burden (%huCD45^+^) in blood from cardiac puncture **(E)**, the bone marrow (BM) **(F)**, and spleen **(G)** over time. The BM includes measurements from left and right femurs.

In an effort to understand the differences in responses to ZV shown by PDXs with equivalent expression levels of ROR1, we assessed the total antibody and conjugated payload plasma levels and ROR1 receptor occupancy in NSG mice engrafted with ALL‐7, a PDX with high ROR1 expression that responded well to ZV in vivo. The plasma concentrations of ZV (conjugated payload) and UC‐961 (total antibody originating from ZV) were quantified in blood from cardiac punctures of mice after a single treatment of ZV (either 2.5 or 5 mg/kg) at multiple time points (Figure 3A). The median total antibody concentration of UC‐961 in plasma peaked at 2 h posttreatment, reaching 47.9 µg/mL (2.5 mg/kg) and 81.7 µg/mL (5 mg/kg). By 48 h, the concentration of the total antibody had decreased to 4.3 µg/mL (2.5 mg/kg) and 18.4 µg/mL (5 mg/kg). A similar profile was observed on measuring plasma ZV (conjugated payload), with median concentrations also peaking at 2 h posttreatment, reaching 51.1 µg/mL (2.5 mg/kg) and 101 µg/mL (5 mg/kg) and decreasing to 3.9 µg/mL (2.5 mg/kg) and 14.9 µg/mL (5 mg/kg) at 48 h. The concentrations of both ZV and UC‐961 were below the lower limit of detection at either 72 h (2.5 mg/kg) or 168 h (5 mg/kg).

To measure receptor occupancy, we analyzed blood from the same cardiac punctures as those used in the PK study. ZV bound rapidly to ROR1^+^ ALL‐7 cells, with >98% median receptor occupancy within 2 h of treatment at both the 2.5 and 5 mg/kg doses (Figure 3B). Near‐maximal receptor occupancy was maintained for at least 48 h at the 2.5 mg/kg dose before measuring below 0% by 72 h, and for 96 h at the 5 mg/kg dose before measuring below 0% by 168 h. Using the ROR1‐targeting antibody 4A5, which is non‐competitive with UC‐961 and ZV, we observed a considerable decrease in the proportion of ROR1^+^ (%ROR1^+^) leukemia cells after 24 h of ZV treatment at both the 2.5 mg/kg (median 18.8%; Figure 3C) and 5 mg/kg (median 14.9%; Figure 3D) doses compared with the baseline control at the 0‐h time point (median 96.3%). Treatment with both doses of ZV was followed by recovery of %ROR1^+^ ALL‐7 leukemia cells in the blood that coincided with the return of ROR1 receptor occupancy to baseline levels. In contrast, the overall leukemic burden (%huCD45^+^) in the cardiac puncture samples remained relatively high across the measured time points (2.5 mg/kg, median 39.4%–58.8%; 5 mg/kg, median 25.3%–53.9%) (Figure 3E), indicating that a substantial ROR1^−^ population persisted in the presence of ZV, despite ALL‐7 being characterized as 96.3% ROR1^+^ (Figure 3C,D) at baseline. We simultaneously measured leukemic burden in the BM and spleen, and observed a progressive decrease in the %huCD45^+^ leukemia cells in the BM over the measured time points, reaching their lowest median values at 168 h (2.5 mg/kg, 74.6%; 5 mg/kg, 44.9%, Figure 3F). However, ZV had minimal impact in the spleen, with the %huCD45^+^ cells reaching a nadir of 93.6% at 72 h (2.5 mg/kg) and 87.2% at 168 h (5 mg/kg, Figure 3G).

### In vivo efficacy of ZV following treatment with the induction‐type regimen VXL

Given that the recently initiated Phase 1/2 clinical trial of ZV includes relapsed/refractory pediatric B‐ALL patients who would have failed conventional combination chemotherapy (LIGHTBEAM‐U01‐Substudy 01 A, ClinicalTrials.gov Identifier NCT06395103), we evaluated the efficacy of ZV immediately after treatment with an induction‐type regimen (VXL) previously optimized in our lab.^30^ ALL‐7 was previously shown to be relatively resistant to the 3‐drug VXL regimen compared with other ALL PDXs.^30^ The regimen of VXL (2 weeks), followed by ZV (once weekly × 3 weeks), was well tolerated in naïve NSG mice receiving up to 5 mg/kg ZV (Supporting Information S1: Figure S6).

**Figure 4:**
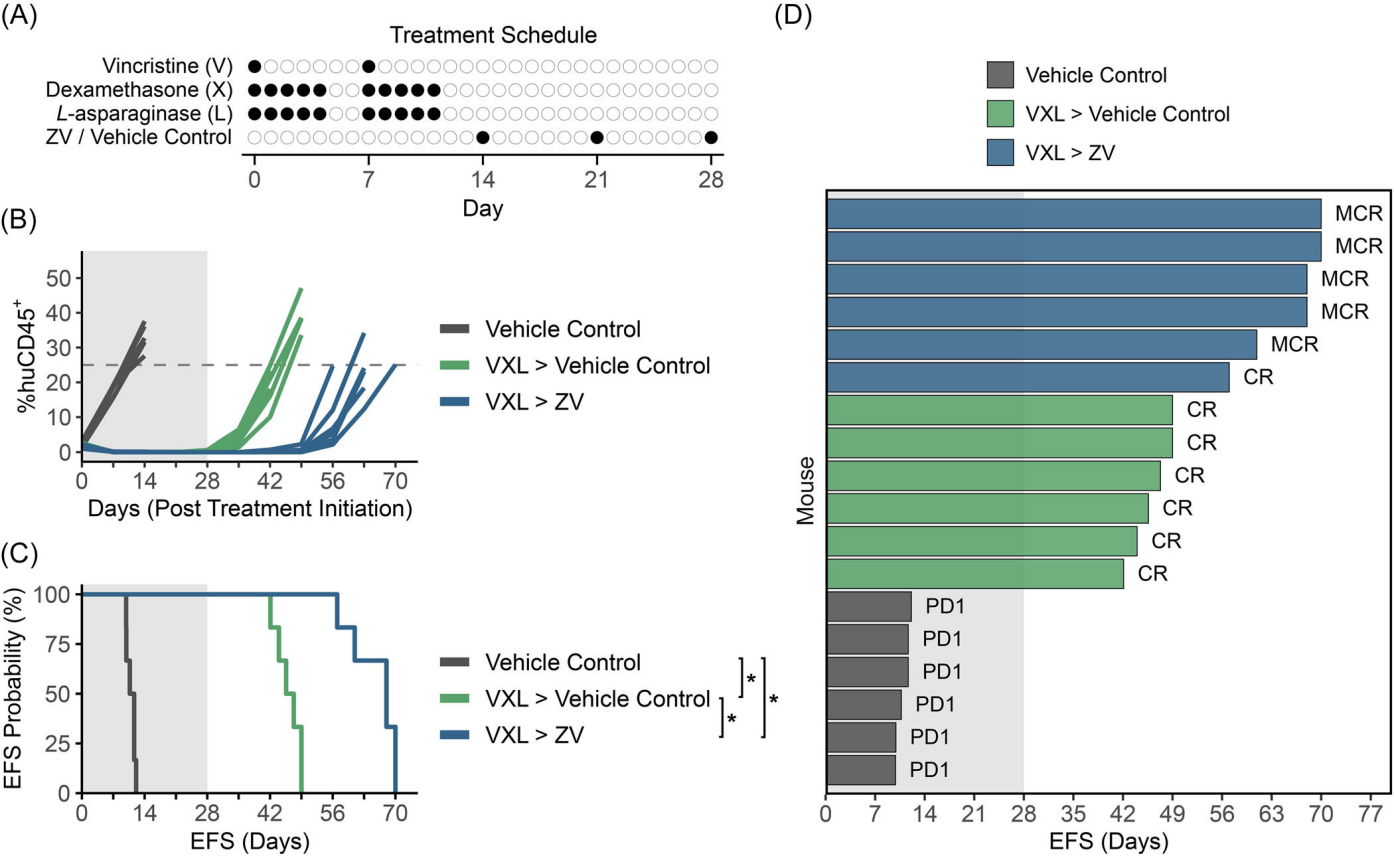
**In vivo efficacy of ZV after treatment with VXL**. **(A)** Schematic of the experimental design for mice administered VXL, followed by ZV or vehicle control (*n* = 6). **(B, C)** Leukemia engraftment **(B)** and Kaplan–Meier survival **(C)** plots after treatment initiation with vehicle control (black), VXL, followed by a ZV vehicle control (VXL > Vehicle Control, green), or VXL, followed by ZV (VXL > ZV, blue). The dotted horizontal line shows the threshold for an event (25% huCD45^+^ cells). Groups were compared with the Peto–Peto modification of the Gehan–Wilcoxon test (BH‐adjusted P = 0.001 for all pair‐wise comparisons, denoted with an asterisk). **(D)** Swimmer plot showing individual mouse EFS in days after treatment initiation and with ORMs shown at the end of each bar. The shaded area represents the treatment window. CR, complete response; MCR, maintained complete response; PD1, progressive disease 1.

For the combination efficacy study, mice engrafted with ALL‐7 were assigned to one of three treatment arms: (1) treatment with the VXL vehicle control for two weeks, followed by weekly administration of the ZV vehicle control for three weeks (Vehicle Control); (2) treatment with VXL for two weeks, followed by weekly administration of the ZV vehicle control over three weeks (VXL > Vehicle Control); and (3) treatment with VXL for 2 weeks, followed by weekly administration of 5 mg/kg ZV over 3 weeks (VXL > ZV) (Figure 4A). Treatment with VXL > Vehicle Control resulted in a greater median EFS compared to the Vehicle Control group (T‐C, 35.3 days; T/C, 4.2; adj. P = 0.001; Figure 4B,C and Table 2). Treatment with VXL > ZV resulted in the greatest extension of EFS (T‐C, 56.8 days; T/C, 6.1; adj. P = 0.001). Both VXL > Vehicle Control (median CR) and VXL > ZV (median MCR) elicited objective responses in ALL‐7 (Figure 4D). Furthermore, ZV remained effective after exposure to VXL, as the difference in EFS when comparing VXL > Vehicle Control and VXL > ZV (21.6 days, Table 2) was similar to the EFS T‐C observed in the single‐agent efficacy study for mice with ALL‐7 treated with 5 mg/kg ZV (19.7 days, Table 1). Complete summary data for the in vivo combination efficacy experiments are included in Supporting Information S2: Table S6.

**Table 2:** Summary of the in vivo results from the combination efficacy study.

Model	Treatment Group	Median EFS	EFS T‐C	EFS T/C	BH‐adjusted p‐value	Median ORM
ALL‐7	Vehicle Control	11.2				
	VXL > Vehicle Control	46.4	35.3	4.2	0.001	CR
	VXL > ZV	68.0	56.8	6.1	0.001	MCR
	VXL > Vehicle Control vs VXL > ZV		21.6	1.5	0.001	

*Note*: Mice were engrafted with ALL‐7 and treated with the vehicle control, VXL, followed by the vehicle control, or VXL, followed by ZV. EFS of treatment groups was compared using the Peto–Peto modification of the Gehan–Wilcoxon test, and P‐values were adjusted with the Benjamini–Hochberg procedure.

Abbreviations: CR, complete response; EFS, event‐free survival; MCR, maintained complete response; ORM, objective response measure; T‐C, treatment minus control; T/C, treatment divided by control.

## DISCUSSION

In this study, we investigated the therapeutic efficacy of the ROR1‐targeting ADCs ZV and VLS‐211 against a panel of 7 BCP‐ALL PDXs. We demonstrate that both ZV and VLS‐211 delayed disease progression in multiple PDXs, but had limited capacity to induce objective responses. Expression of the antigen was required but not sufficient for a response to either ADC, and we did not observe a correlation between ROR1 expression and EFS or objective responses for either ZV or VLS‐211. It is unclear if the presence of different driver fusions would obscure any correlation, as response is likely to be influenced by molecular subtype. However, it was noteworthy that for the patient‐matched PDXs (ALL‐82, at diagnosis and ALL‐83, at relapse), there was both higher ROR1 cell surface expression and an improved response in the PDX derived after relapse. ZV was generally well tolerated at the doses tested but toxicity was a concern for VLS‐211, particularly at the highest tested dose. Given that treatment with the naked antibody UC‐961 showed no activity across all evaluated PDXs, our data suggest that any toxicity observed from treatment with ZV or VLS‐211 is associated with their respective payloads, a phenomenon commonly observed in other ADC studies.^33^

Although the subtypes with high ROR1 expression across the 90 PDXs were represented in the in vivo efficacy study, several subtypes including MEF2D‐rearranged and PAX5‐altered B‐ALL were not evaluated despite high expression in many patients. The panel tested included two PDXs with high expression of *ROR1* that are classified as high risk: one harboring a *TCF3*::*HLF* fusion (ALL‐7) and another with a *BCR*::*ABL1* fusion (ALL‐4). Leukemia with *TCF3*::*HLF* fusions is highly chemo‐resistant,^34^ though recent reports have documented that leukemia cases with the *TCF3*::*HLF* fusion are responsive to CD19‐targeting agents like blinatumomab and to CD19/CD22 CAR T‐cells.^35^, ^36^ It is noteworthy that the PDX harboring this fusion was among the top responders and had the highest expression of *ROR1* in our panel. Similarly, *ROR1* expression was also the highest in *TCF3*::*HLF* patient samples analyzed in the TARGET study. Together, the data suggest that this subtype may be more sensitive to ZV or other immunotherapeutic approaches that target ROR1. In contrast, ZV and VLS‐211 elicited a poor response in ALL‐4, despite having high expression of ROR1. This observation raises the possibility that the lack of response could be attributable to resistance mechanisms specific to the payloads of these ADCs or, alternatively, resistance to this class of drugs through other mechanisms, such as reduced receptor internalization.^37^ However, because of a lack of PDXs sharing the same rearrangement, we cannot confidently generalize these findings across entire subtypes.

Despite promising preclinical results, the clinical translation of ADCs for the treatment of ALL has achieved limited success. To date, inotuzumab ozogamicin, which targets CD22, remains the sole ADC approved for the treatment of ALL.^38^ One reason for the difficulties in translating preclinical ADC results to the clinic is the common use of doses for preclinical testing that exceed what is realistically achievable in humans.^38^ Current best practice for preclinical testing of ADCs is to match the preclinical testing dose (in mg/kg) with the tolerable dose observed in patients (in mg/kg).^39^, ^40^ The doses selected for ZV (2.5 and 5.0 mg/kg) align with the ZV single‐agent dose used in clinical testing of ZV,^21^, ^22^, ^41^ albeit with the preclinical schedule of administration (weekly × 3) being more frequent than the every‐3‐weeks clinical administration schedule. A comparison of the PK parameters for ZV in our preclinical models to those observed in patients shows a close match for *T*_max_ and *C*_max_, with these being close to 2 h and 50 µg/ml, respectively.^41^ The ZV half‐life was shorter in preclinical models compared to the approximately 4‐day half‐life observed in patients with solid tumors. This difference could indicate that the preclinical PK was performed in animals engrafted with ROR1‐expressing human leukemia cells, which could act as an antigen sink and thereby reduce ZV circulating half‐life.^42^

Loss of cell surface antigen expression is commonly observed in response to ADCs and other antibody‐related treatments. For example, antibody‐induced downregulation of the receptor tyrosine kinases insulin‐like growth factor receptor 1 (IGF‐1R) and MET occurs in response to treatment with antibodies targeting these proteins.^43^, ^44^ ROR1 also internalizes in response to antibody binding, with resulting lower levels of ROR1 at the cell surface.^16^ In ALL‐7, treatment with ZV led to a rapid decrease in ROR1^+^ cells, followed by a return to baseline within 96 h. The speed and magnitude of this shift, coupled with the relatively stable leukemic burden, point toward internalization and degradation of ROR1 rather than the negative selection of a fixed ROR1^+^ population. The delayed return of ROR1 expression on leukemia cells likely reflects the combined effects of the kinetics of ROR1 synthesis as well as the time course for reduction of ZV levels. While the data are consistent with ZV delivering the payload to its target, future studies will investigate if this is true for the other PDXs that did not respond as well as ALL‐7.

Due to the prominent role of ROR1 in hematological cancers, many therapeutic approaches including monoclonal antibodies, BiTEs, and CAR T‐cell therapies have been developed to target this receptor.^14^ This has led to clinical trials investigating the BiTE NVG‐111 for multiple ROR1^+^ malignancies, including chronic lymphocytic leukemia, small lymphocytic lymphoma, mantle cell lymphoma, follicular lymphoma, diffuse large B‐cell lymphoma, non‐small cell lung cancer, and melanoma (ClinicalTrials.gov number, NCT04763083). Furthermore, several CAR T‐cell therapy trials targeting ROR1 were initiated (ClinicalTrials.gov numbers, NCT05694364, NCT05588440, and NCT02706392); however, the latter two trials have since been terminated.^45^ Unlike BiTEs and CAR T‐cell therapy, ADCs do not depend on a functioning immune system and may be an optimal choice for combining with chemotherapy that is often toxic to the patient's immune cells.

Given the modest activity of ZV and VLS‐211 along with the high standards for new agents entering the clinic for treating B‐ALL, both agents are unlikely to provide significant benefit to most ALL patients. Nevertheless, they may prove valuable for treating high‐risk cases with high ROR1 expression for which conventional therapies have failed, and treatment options remain limited. Notably, ZV demonstrated a stronger therapeutic effect in a PDX derived from a relapsed patient (ALL‐83) compared to one from the same patient at diagnosis (ALL‐82). Furthermore, ZV retained its potency in a PDX model (ALL‐7) following exposure to an induction‐type regimen. Both findings are particularly relevant for pediatric patients with relapsed/refractory B‐ALL enrolled in the LIGHTBEAM‐U01‐Substudy 01 A clinical trial. Furthermore, other combinatorial strategies (e.g., ZV administered simultaneously with traditional chemotherapy) have not been explored and may be another avenue to pursue in the clinic. Taken together, ZV and other ROR1‐targeting ADCs may have utility in the relapsed/refractory setting of ROR1^+^ B‐ALL; however, developments to improve their potency may be required and further evaluation of their efficacy in combination with other commonly used therapies in ALL is warranted.

## AUTHOR CONTRIBUTIONS

**Christopher M. Smith**: Data curation; software; formal analysis; visualization; methodology; writing—original draft; writing—review and editing. **Ben Watts**: Data curation; formal analysis; investigation; visualization; methodology; writing—original draft; writing—review and editing. **Kathryn Evans**: Conceptualization; data curation; formal analysis; validation; investigation; methodology; writing—review and editing. **Hansen Kosasih**: Formal analysis; visualization; methodology; writing—review and editing. **Chelsea Mayoh**: Data curation; software; writing—review and editing. **Stephen W. Erickson**: Formal analysis; visualization; writing—review and editing. **Eric J. Earley**: Formal analysis; visualization; writing—review and editing. **Steven Neuhauser**: Formal analysis; Visualization; Writing—review and editing. **Timothy M. Stearns**: Formal analysis; visualization; writing—review and editing. **Vivek M. Philip**: Formal analysis; visualization; writing—review and editing. **Jeffrey H. Chuang**: Resources; project administration; funding acquisition. **Charles E. de Bock**: Methodology; writing—review and editing. **Edward P. Bowman**: Conceptualization; resources; writing—review and editing. **Katti A. Jessen**: Conceptualization; resources; writing—review and editing. **Emily L. Jocoy**: Project administration; writing—review and editing. **Carol J. Bult**: Resources; project administration; writing—review and editing; funding acquisition. beverly a teicher: resources; project administration; writing—review and editing. **Malcolm A. Smith**: Conceptualization; resources; project administration; writing—review and editing; funding acquisition. **Richard B. Lock**: Conceptualization; resources; writing—original draft; project administration; writing—review and editing; supervision; funding acquisition.

## CONFLICT OF INTEREST STATEMENT

Edward P. Bowman was employed by Merck Sharp & Dohme LLC, a subsidiary of Merck & Co., Inc., Rahway, NJ, USA, during the course of part of this research work. Katti A. Jessen is a former employee of VelosBio, Inc. All other authors declare no conflict of interest. Charles E. de Bock is an Editor at *HemaSphere*.

## ETHICS STATEMENT

All animal experiments received prior approval from the Animal Care and Ethics Committee of UNSW Sydney (Sydney, New South Wales, Australia).

## FUNDING

This research was funded by grants from the National Cancer Institute (CA199000, CA199222, and CA263963), a fellowship from the National Health and Medical Research Council of Australia (1157871) to RBL, and by the Annie Frida Minna Adams Charitable Trust, the Hillcrest Foundation, and The Alma Hazel Eddy Trust. Children's Cancer Institute Australia is affiliated with UNSW Sydney and The Sydney Children's Hospitals Network.

## Supporting information

Supporting File 1.

Supporting File 2.

## Data Availability

The data that support the findings of this study are available from the corresponding author upon reasonable request. The data generated in this study are available upon request from the corresponding author.
