# Supplementary material for: Preclinical evaluation of the ROR1‐targeting antibody–drug conjugates zilovertamab vedotin and VLS‐211 against B‐cell ALL patient‐derived xenografts
Source: Hemasphere. 2026 Jul 22;10(7):e70438. doi: 10.1002/hem3.70438 (PMC13390736; doi:10.1002/hem3.70438)
Supplement: Supplementary file 1 — Supporting File 1. [file HEM3-10-e70438-s001.docx]

**Preclinical evaluation of the ROR1 targeting antibody-drug conjugates zilovertamab vedotin and VLS-211 against B-cell ALL patient-derived xenografts**

**SUPPLEMENTARY INFORMATION**

**SUPPLEMENTARY METHODS**

**Statistical Methods for Evaluating Treatment Response**

The exact time-to-event is estimated by interpolating between the measurements directly preceding and following the event, assuming log-linear growth. Differences in event-free survival (EFS) between experimental groups (e.g., treated vs controls) are tested using the *G^ρ^* test of Harrington & Fleming (*Biometrika* 69:553-566, 1982; *α* = 0.05, two-sided alternative) with *ρ* = 1, which is equivalent to the Peto & Peto modification of Gehan-Wilcoxon.

The *objective response measure* (ORM) categories are progressive disease (PD, which is subdivided into progressive disease without and with growth delay, PD1 and PD2 respectively, defined only for treated mice), stable disease (SD), partial response (PR), complete response (CR), and maintained complete response (MCR).

- PD when %huCD45^+^ is never < 1% during study period and the mouse reaches event (%huCD45^+^ > 25%) at some point during the study period (42 days),
- PD1 when PD, and the time to event is ≤ 200% of the median time-to-event in the control group,
- PD2 when PD, but the time-to-event is > 200% of the median time-to-event in control group,
- SD when %huCD45^+^ is never < 1% and the mouse never reaches event during the study period,
- PR when %huCD45^+^ is < 1% at least once during the study period, but not CR,
- CR when %huCD45^+^ is < 1% for at least 2 consecutive weekly readings during the study period, regardless of whether event is reached at a later time-point, and
- MCR when %huCD45^+^ is < 1% for at least 3 consecutive weekly readings at any time after treatment has been completed.

The overall group response is determined by the median response among evaluable mice as follows: each mouse is assigned a score from 0 to 10 based on their response, where PD1 = 0, PD2 = 2, SD = 4, PR = 6, CR = 8, and MCR = 10. The median for the group then determines the overall response. If the median score is half-way between an objective response number category, the objective response is assigned to the lower response category (e.g., an objective response score of 9 is scored CR). Studies in which toxicity is greater than 25%, or in which the control group is not SD or worse, are considered unevaluable and are excluded from analysis. Treatment groups with PR, CR, or MCR are considered to have had an objective response. Agents inducing objective responses are considered highly active against the tested line, while agents inducing SD or PD2 are considered to have intermediate activity, and agents producing PD1 are considered to have a low level of activity against the tested line.

Summary tables for efficacy experiments will summarize results for each experimental group (e.g., treated vs controls) and include the following columns:

- PDX, the alphanumeric code for the tumor model,
- Grp, experimental group, usually C (Control) or T (Treatment)
- *N*, the total number of mice entering experiment,
- *N_d_*, the number of mice experiencing toxic death,
- *N_x_*, the number of additional mice excluded from analysis,
- *N_a_*, the number of mice in analysis,
- *N_ev_*, the number of events,
- KMmed, the Kaplan-Meier estimate of median time-to-event (days),
- EFS T-C, the difference in median time-to-event (days) between T and C groups,
- EFS T/C, the ratio of median time-to-event between T and C groups,
- EFS *p*-value, computed using the Gehan-Wilcoxon test,
- Baseline %huCD45^+^ mean+SD, the mean + standard deviation of the per-mouse baseline %huCD45^+^,
- %huCD45^+^ *p*-value between groups A and B at baseline, computed using Wilcoxon rank sum test
- min %huCD45^+^ mean±SD, the mean ± standard deviation of the per-mouse minimum %huCD45^+^,
- min %huCD45^+^ T/C value
- %huCD45^+^ *p*-value, computed using Wilcoxon rank sum test,
- Fold change in %huCD45^+^ from baseline to minimum post treatment (medium plus interquartile range)
- One column for each category of objective response (i.e. PD, PD2, etc), showing the number of mice in each category,
- Resp rate, the response rate, defined as the percentage of mice having PR or better, and

Med resp, the median response evaluation.

**Total human IgG assay in mouse plasma**

The concentration of “total human IgG” in mouse plasma of the test article was measured by a bioanalytical method using an ECL-based immunoassay platform. The lower limit of quantitation (LLOQ) of test article in mouse plasma is 13.7 ng/mL. Briefly, 96-well flat-bottom MSD Streptavidin Gold multi-array plates were blocked with 5% bovine serum albumin (BSA) in PBS followed by coating with biotinylated mouse anti-human Ig kappa light chain antibody in Modified ELISA Diluent buffer (MED) (0.5% BSA [wt/v], 0.05% Tween 20 [v/v], 0.25% CHAPS [wt/v], 5 mM EDTA in PBS at pH 7.4). The detection reagent was a Sulfo-Tagged mouse anti-human IgG CH2 domain antibody in MED. Standards, controls, and sample dilutions followed by detection reagent were added in between sequential wash steps and incubations. After a final wash, MSD 1X Read Buffer T with Surfactant was added and ECL was measured on MSD plate reader, Meso Sector S600. The resulting ECL signal was proportional to the concentration of the test article in the samples. After a minimum required dilution of 1:20 with MED, samples were quantitatively measured by comparison with a standard curve of the test article with a range of 0.69 to 500 ng/mL.

**Conjugated payload assay in mouse plasma**

The concentration of “payload conjugated to antibody” in mouse plasma was quantified by affinity capture, on-cartridge proteolytic release, and LC–MS/MS. Calibration standards were prepared by spiking ZV into blank mouse plasma. Briefly, biotinylated anti‑human IgG F(ab')2 (cat #109‑066‑088, Jackson ImmunoResearch) was loaded onto 5 µL streptavidin cartridges (cat # G5496‑60010, Agilent) using an AssayMap Bravo (Agilent). Diluted plasma samples and ADC standards in blank mouse plasma were applied to the cartridges, which were washed on the platform to remove unbound matrix. Conjugated payload was released by on‑cartridge digestion with freshly activated papain (2 mg/mL in 100 mM Tris‑HCl, pH 8.0, 2 mM cysteine; cat #10108014001, Roche) incubated at 37 °C for 15 min, and eluates were collected. Eluates were mixed 1:4 (v/v) with acetonitrile containing 0.1% formic acid and imipramine as internal standard, vortexed, and centrifuged at 4,200 × g for 10 min. Supernatants were analyzed on a Waters ACQUITY UPLC HSS T3 column (1.8 µm, 2.1 × 50 mm) at 40 °C with mobile phases A (0.1% formic acid in water) and B (0.1% formic acid in acetonitrile). The flow rate was 750 µL/min with the following gradient: 95% A/5% B (initial, hold to 0.25 min), linear to 5% A/95% B at 1.75 min (hold to 2.16 min), return to initial at 2.17 min and re‑equilibration to 3.00 min total run time. Detection was by AB SCIEX API 6500 LC‑MS/MS with TurboIonSpray using positive‑mode MRM; data were acquired with Analyst 1.7.2 and quantified in MultiQuant 3.0 against calibration standards using linear 1/x^2 weighting.

**SUPPLEMENTARY FIGURES**


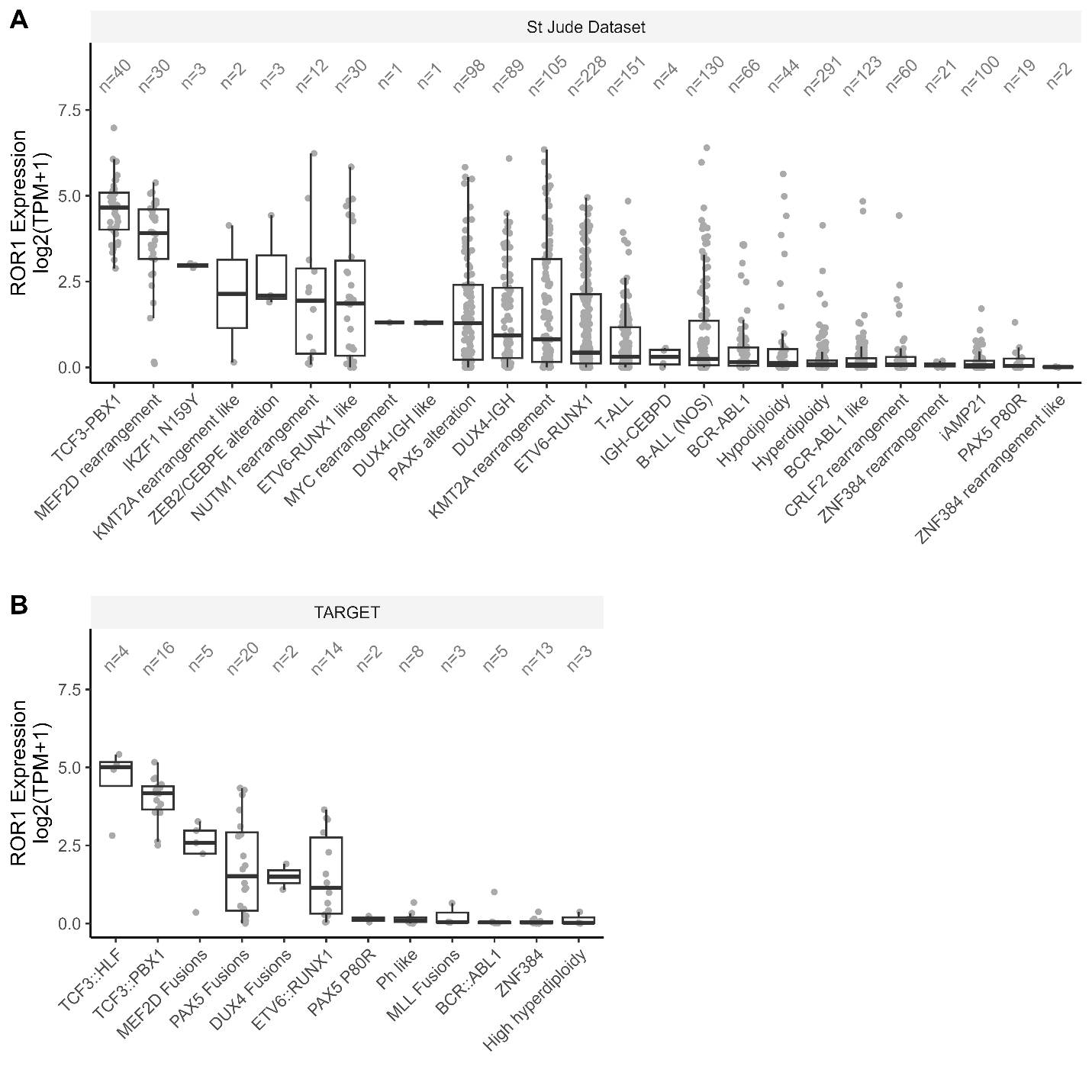


**Supplementary Figure S1.** Expression of *ROR1* mRNA in ALL patients from the St. Jude dataset (A) and a subset of patients from the TARGET study (B). Expression shown as the log_2_ transformation of transcripts per million (TPM) with an added pseudocount of 1. Dots show expression of individual patients and box plots show group medians.

**
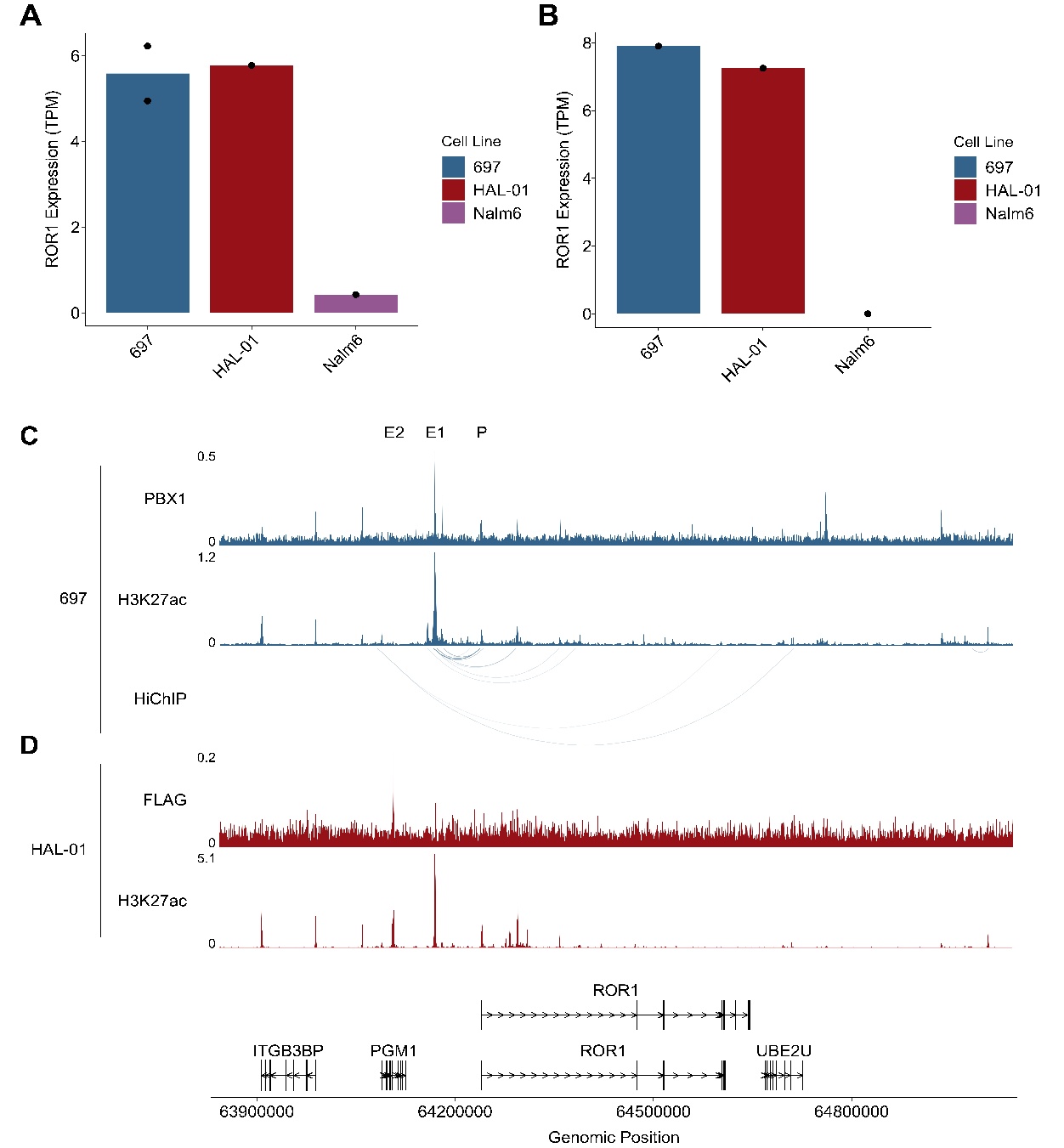
**

**Supplementary Figure S2.** *ROR1* expression and genomic view of the *ROR1* locus in ALL cell lines. (A, B) *ROR1* RNA expression in 697 (*TCF3*::*PBX1*, blue), HAL-01 (*TCF3*::*HLF*, red), and Nalm6 (no fusion, purple) cell lines from the FORALL (A) and Tamai et al (B) datasets (n=1, except 697 in FORALL dataset where n=2). Black dots show biological replicates and bars show median expression. (C, D) ChIP-seq and HiChIP tracks covering the *ROR1* locus. For ChIP-seq, the y-axis shows the normalized counts per million, smoothed with a rolling averaged window of 10 bp. Localization of the fusion protein was determined using PBX1 for 697 (blue) or FLAG for HAL-01 (red) expressing FLAG-tagged *TCF3*::*HLF*. Putative *ROR1* promoter is labelled as P. Active enhancer sites (labelled E1 and E2) were determined by presence of the H3K27ac histone mark. HiChIP data for 697 are visualized as arcs connecting significant interacting genomic regions (q-value < 0.05). The darker lines indicate a stronger association (smaller q-value). Gene annotations and genomic coordinates (chromosome 1, human genome build hg19) are shown at the bottom of the figure.


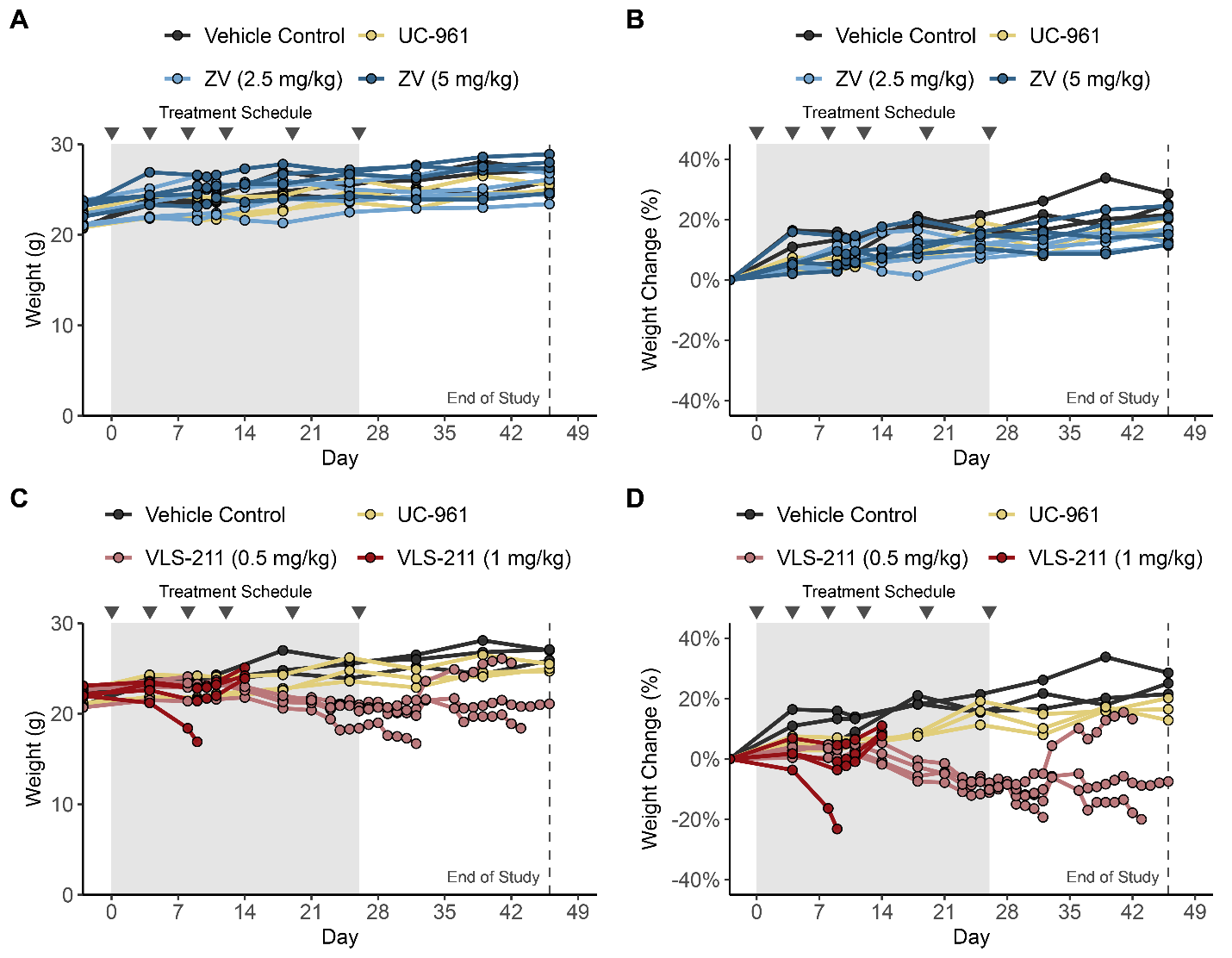


**Supplementary Figure S3.** Tolerability of ZV and VLS-211 in naïve NSG mice. ZV at 2.5 mg/kg (light blue) and 5 mg/kg (dark blue), VLS-211 at 0.5 mg/kg (pink) and 1 mg/kg (red), UC-961 (yellow) or vehicle control (black) were scheduled for 6 total treatments, administered via intravenous injection every four days, but were altered to once a week from the fourth treatment due to toxicity. Weight (A, C) and the % weight change (B, D) for ZV (A-B) and VLS-211 (C-D). The shaded area represents the treatment window, and the black triangles represent treatment administration.


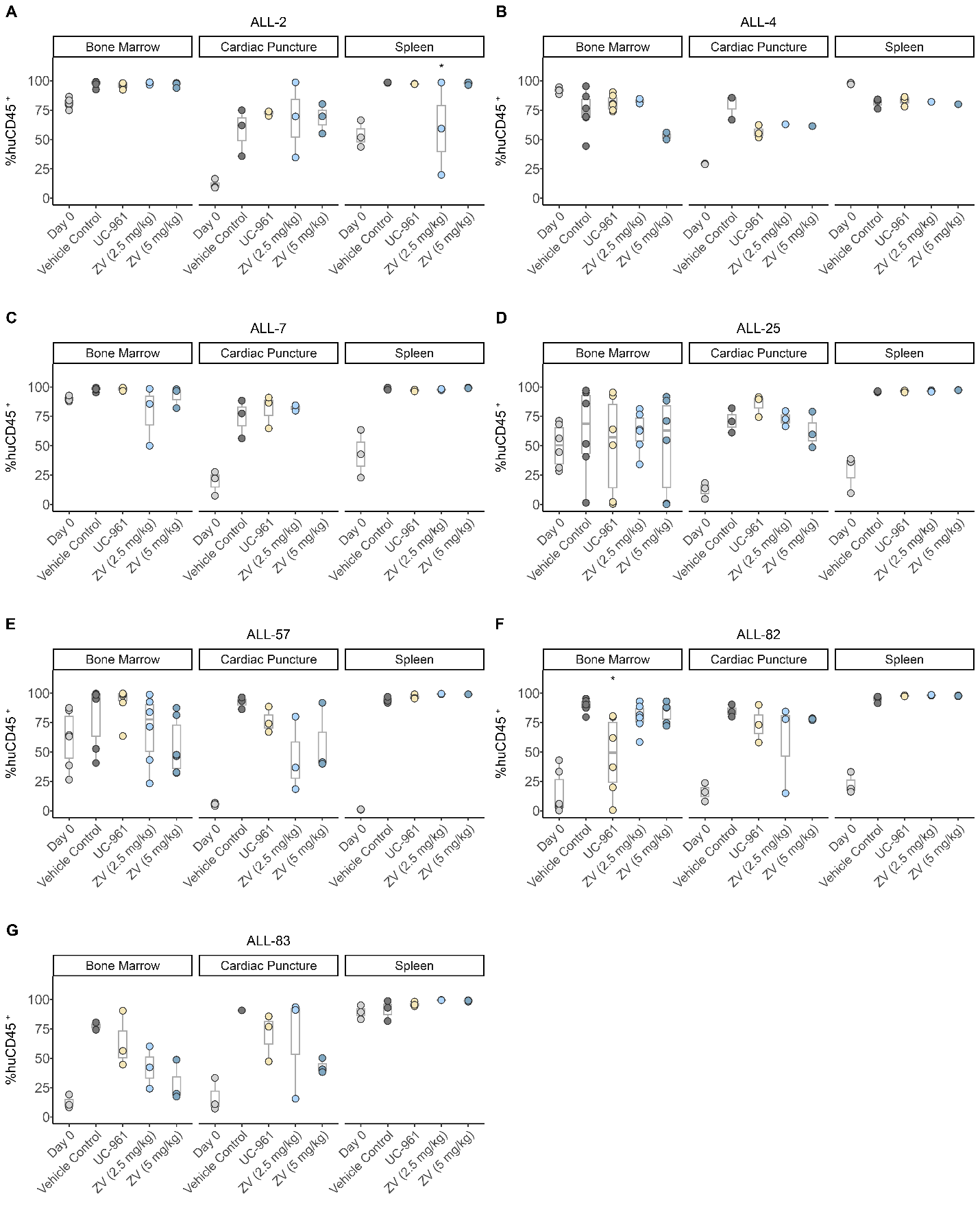


**Supplementary Figure S4.** **Leukemic infiltration in hematolymphoid organs following treatment with ZV.** Infiltration of leukemia cells was measured by the enumeration of human CD45^+^ (%huCD45^+^) cells in BM, cardiac puncture and spleen samples at event or day 28 (whichever occurred first). Dots show values for individual mice and boxes show group medians. For comparison, baseline (Day 0), vehicle control and UC-961 groups are reproduced in Supplementary Figures S4 and S5. Asterisks (*) are shown for significant differences between treatment groups and vehicle control (*p* < 0.05).


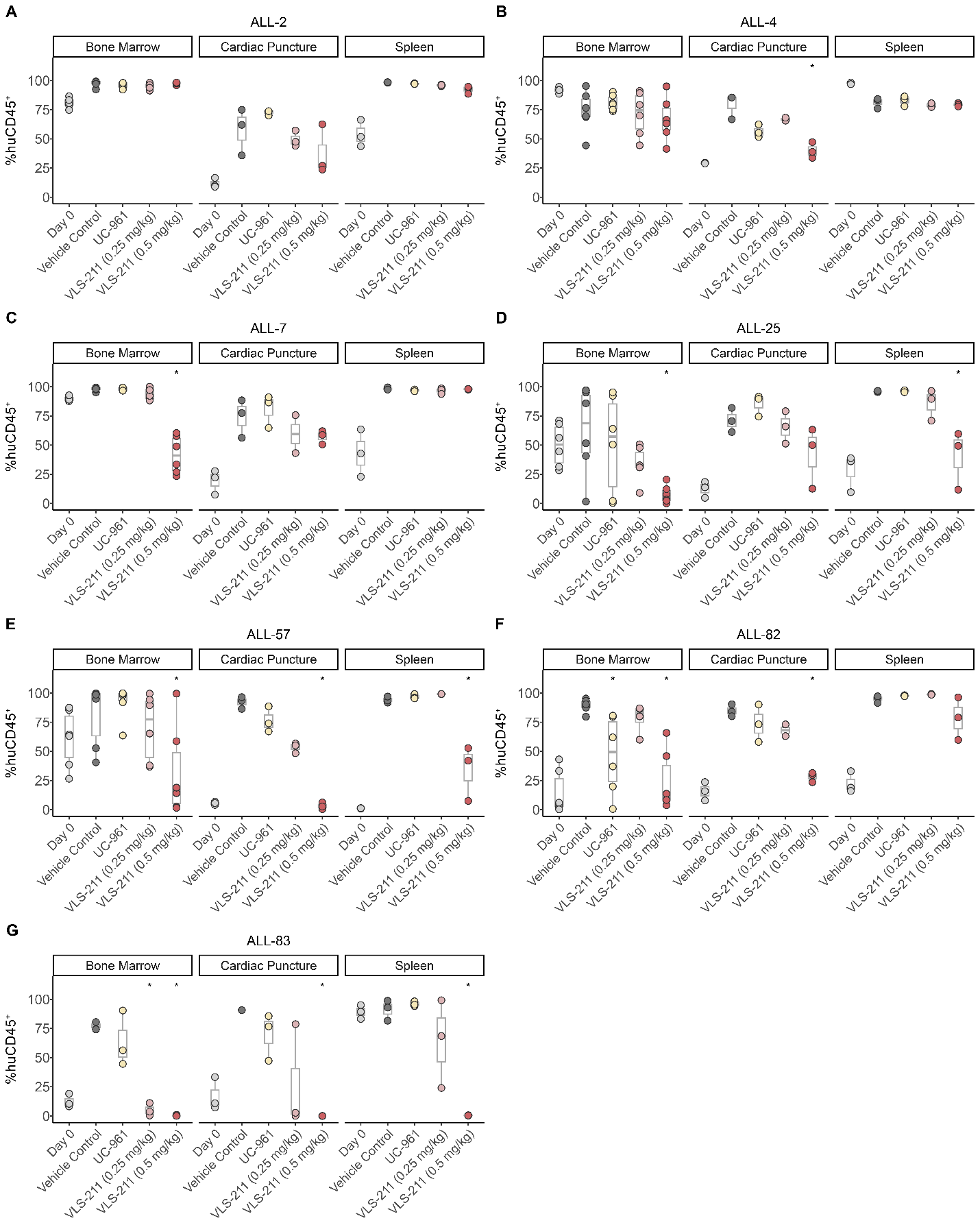


**Supplementary Figure S5.** **Leukemic infiltration in hematolymphoid organs following treatment with VLS-211.** Infiltration of leukemia cells was measured by the enumeration of human CD45^+^ (%huCD45^+^) cells in BM, cardiac puncture and spleen samples at event or day 28 (whichever occurred first). Dots show values for individual mice and boxes show group medians. For comparison, baseline (Day 0), vehicle control and UC-961 groups are reproduced in Supplementary Figures S4 and S5. Asterisks (*) are shown for significant differences between treatment groups and vehicle control (*p* < 0.05).


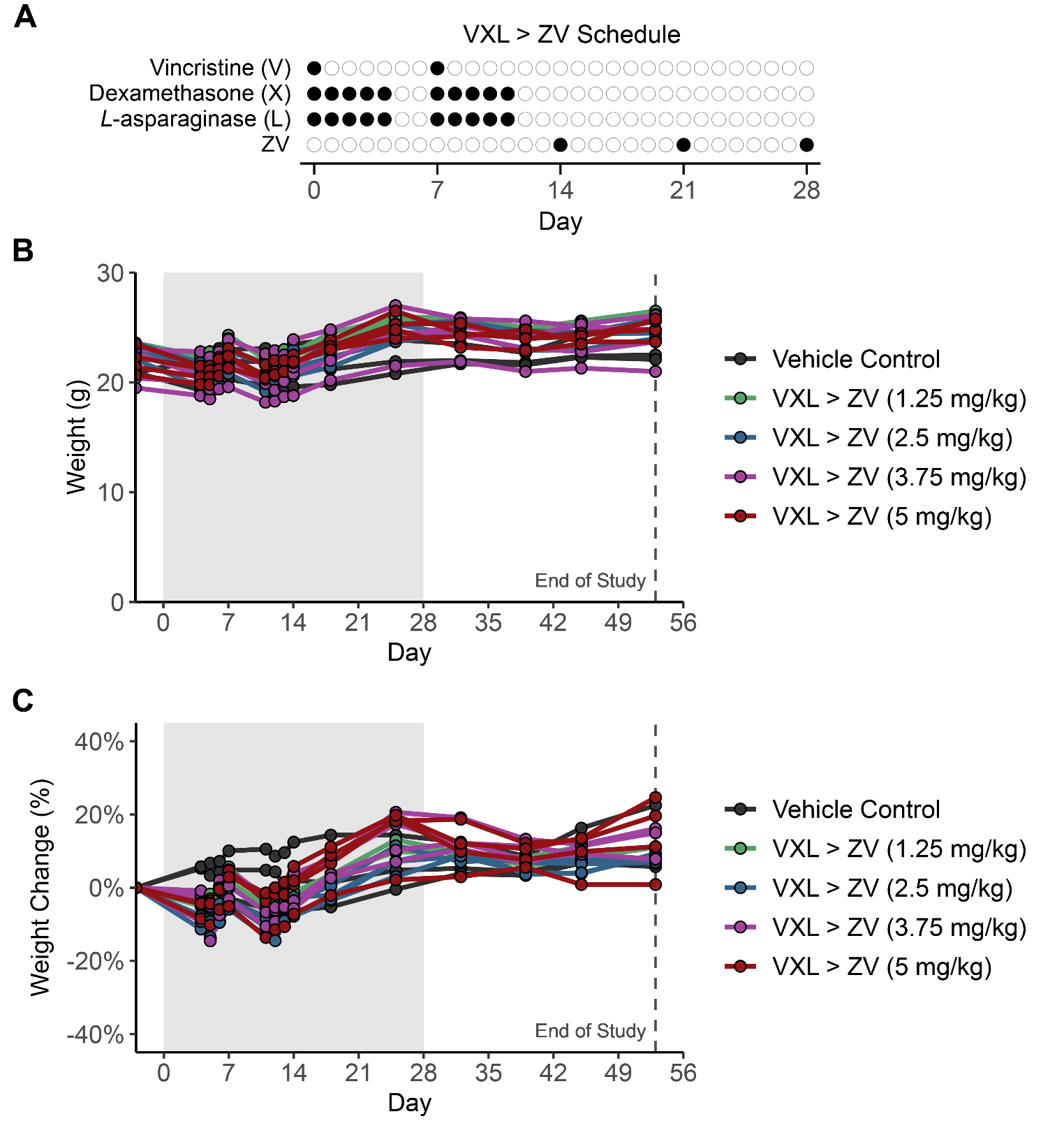


**Supplementary Figure S6.** Tolerability of the sequential treatment of an ALL induction-type regimen followed by ZV (VXL > ZV) in naïve NSG mice. (A) Treatment schedule. (B-C) Weight (B) and the % weight change from baseline (C) after treatment with VXL followed by 1.25 mg/kg (green), 2.5 mg/kg (blue), 3.75 mg/kg (purple) or 5 mg/kg (red) of ZV. The shaded area represents the treatment window.

**SUPPLEMENTARY TABLES**

Supplementary Tables are available as a separate file.
